# Targeted protein degradation with bifunctional molecules as a novel therapeutic modality for Alzheimer's disease & beyond

**DOI:** 10.1016/j.neurot.2024.e00499

**Published:** 2024-12-04

**Authors:** C. Alexander Sandhof, Heide F.B. Murray, M. Catarina Silva, Stephen J. Haggarty

**Affiliations:** Department of Neurology, Precision Therapeutics Unit, Chemical Neurobiology Laboratory, Center for Genomic Medicine, Massachusetts General Hospital and Harvard Medical School, Boston, MA 02114, USA

**Keywords:** Tau, Alzheimer's disease, Drug discovery, Targeted protein degradation, Precision therapeutics

## Abstract

Alzheimer's disease (AD) is associated with memory and cognitive impairment caused by progressive degeneration of neurons. The events leading to neuronal death are associated with the accumulation of aggregating proteins in neurons and glia of the affected brain regions, in particular extracellular deposition of amyloid plaques and intracellular formation of tau neurofibrillary tangles. Moreover, the accumulation of pathological tau proteoforms in the brain concurring with disease progression is a key feature of multiple neurodegenerative diseases, called tauopathies, like frontotemporal dementia (FTD) where autosomal dominant mutations in the tau encoding *MAPT* gene provide clear evidence of a causal role for tau dysfunction. Observations from disease models, post-mortem histology, and clinical evidence have demonstrated that pathological tau undergoes abnormal post-translational modifications, misfolding, oligomerization, changes in solubility, mislocalization, and intercellular spreading. Despite extensive research, there are few disease-modifying or preventative therapeutics for AD and none for other tauopathies. Challenges faced in tauopathy drug development include an insufficient understanding of pathogenic mechanisms of tau proteoforms, limited specificity of agents tested, and inadequate levels of brain exposure, altogether underscoring the need for innovative therapeutic modalities. In recent years, the development of experimental therapeutic modalities, such as targeted protein degradation (TPD) strategies, has shown significant and promising potential to promote the degradation of disease-causing proteins, thereby reducing accumulation and aggregation. Here, we review all modalities of TPD that have been developed to target tau in the context of AD and FTD, as well as other approaches that with innovation could be adapted for tau-specific TPD.

## Introduction

Currently, over 55 million people worldwide [1] suffer from a form of dementia, which is a class of devastating neurodegenerative diseases marked by the progressive loss of cognitive functions. Alzheimer's disease (AD) is responsible for 60–70 ​% of all dementia cases [1], with estimates predicting over 384 million people, i.e., ∼20 ​% of people over 50 years old, not yet having clinical AD but being in the prodromal or preclinical stages of the disease [2]. Due to the progressive loss of autonomy during disease progression, AD patients require a high level of care. This puts an immense burden not only on the health care system but also on families, caregivers, and elderly care facilities, which contributes to an increasing socio-economic burden as life expectancy around the globe rises and populations undergo a demographic shift. AD and several other neurodegenerative diseases are classified as proteinopathies, sharing the common feature of proteins/peptides aggregating into highly structured amyloid fibers and subsequently into macromolecular inclusions, a process that is thought to play an important role in disease pathogenesis [3]. Generally, AD-affected brains have two main types of proteinaceous inclusions that can be detected by either postmortem histopathological analysis or, based upon emerging advances by positron emission tomography (PET) scans of living patients after exposure to a radiolabeled PET tracer that exhibits specificity for a particular misfolded protein. These two types of inclusions are extracellular amyloid plaques, whose main component is a cleavage product of the amyloid-precursor-protein designated amyloid-β (Aβ)-peptide, and intracellular neurofibrillary tangles (NFTs) composed of hyperphosphorylated misfolded tau protein. With the recently approved Aβ targeting immunotherapies Kisunla [4] and Leqembi [5], at last, the first disease-modifying treatments have become available to patients, although at a high cost and not without serious risk of adverse effects, including amyloid-related imaging abnormalities [6]. While clinical trials demonstrated that these antibody-based therapies can reduce amyloid plaques down to baseline levels and marginally slow the progression of AD, the overall progression of the disease was not stopped. Together with the recognition that AD can be quite heterogeneous among populational cohorts and have multiple subtypes [7,8], there remains an urgent need to develop additional and complementary therapeutic options, including tau-targeting therapies that address the other major amyloidogenic components found in AD brains. In this review, we will cover the rapidly evolving field of targeted protein degradation (TPD) with a focus on strategies that either directly and indiscriminately target all tau protein, or that target specific disease-associated pathological forms of tau for degradation. We will also cover selected novel TPD methods that have not yet been applied to tau but could be readily adapted and tested.

## Microtubule-Associated Protein Tau

### Structure and function

**Fig. 1:**
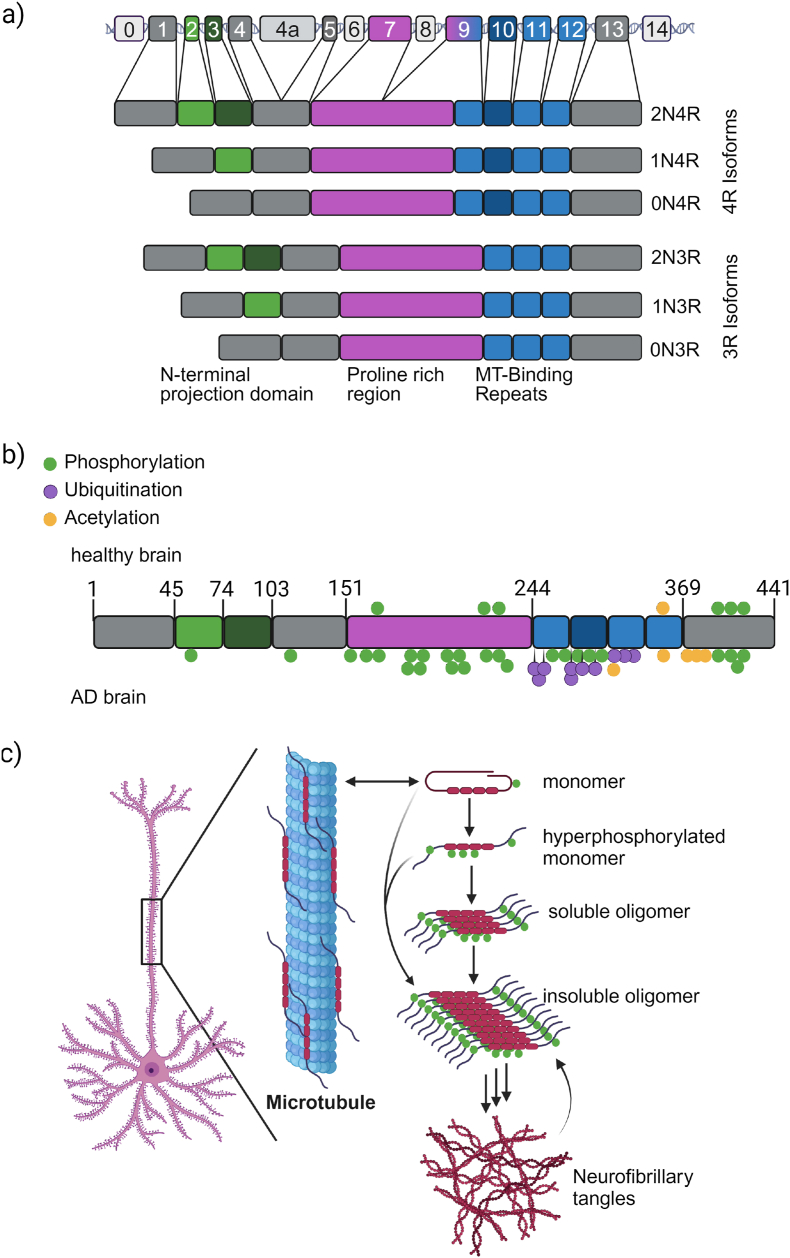
***MAPT*/tau, structure, post-translational modifications, and aggregation in disease. (a)** Structure of the *MAPT* locus and the six CNS splice isoforms expressed in the adult human brain. **(b)** Schematic representation of PTMs identified on 2N4R tau in healthy and late-stage AD brains [31]. Shown PTMs are representative and do not encompass all known ones. **(c)** Axonal tau exists in an equilibrium of microtubule-bound and free monomeric tau in a paperclip fold that prevents aggregation. Hyperphosphorylation disrupts protective interactions between tau's N- and C-termini, and its microtubule-binding repeats and, at the same time, lowers tau's affinity for microtubules. This enables the adoption of a β-sheet rich misfolded structure that allows for the stacking of individual monomers forming soluble oligomers. Subsequent oligomer growth into tau amyloid fibers happens through the incorporation of soluble monomers at the fiber ends. Secondary nucleation along the fiber surface and fiber breakage produces more oligomers fueling the polymerization-like aggregation reaction. Created in BioRender. Sandhof, C. (2024) BioRender.com/h83t121.

The tau protein is encoded by the *MAPT* (microtubule-associated protein tau) gene located on chromosome 17q21. In the central nervous system (CNS), alternative splicing of *MAPT* gives rise to six isoforms of tau, which result from the inclusion or exclusion of exons 2, 3, and 10 [9,10]. Exons 2 and 3 encode inserts in the N-terminal projection domain of tau (0 ​N, 1 ​N, 2 ​N isoforms), with the inclusion of exon 3 dependent on the presence of exon 2; while exon 10 encodes one of four microtubule binding repeat domains (3R or 4R isoforms) (Fig. 1a). Expression of the different tau isoforms is highly regulated and has a strong temporal/developmental component. While 3R and 4R isoforms are expressed roughly at equimolar levels in the adult human brain, with some variation in the number of included N-terminal exons, the fetal brain only expresses the shortest 0N3R isoform. Around the time of birth, a shift in *MAPT* transcript splicing allows for regulated inclusion of exons 2, 3 and 10, initiating 4R tau isoforms expression [11]. Since its identification in 1975 [12], tau has been described as a tubulin-binding factor that regulates the dynamics of microtubule (MT) polymerization. However, its recognized physiological functions have vastly expanded. As elaborating on this subject in depth is beyond this review's scope, we will give only a succinct overview of some key functions and point the reader to articles focusing on that topic [[13], [14], [15]]. Tau belongs to the family of microtubule-associated proteins (MAP) and, together with MAP2, is one of two MAPs expressed in the CNS [16]. While not restricted to neurons [17], the majority of tau in the CNS is expressed by neurons where it is mainly localized in the axonal compartment. Tau is primarily thought to regulate MT dynamics and stability, hence its primary axonal localization. The interaction of tau with MTs is mediated via tau's three (3R) or four (4R) microtubule binding repeats (MTBR, Fig. 1a) [18]. This is a transient interaction with roughly 50 ​% of all tau remaining mobile along the MT fibers [19] to prevent blocking of active transport along the MTs [18]. MT-unbound tau is highly soluble and intrinsically disordered, preventing most structural studies of its native conformation or behavior. However, free monomeric tau is not completely unstructured. Intramolecular interactions between the termini and between the C-terminus and the MTBR promote the formation of a so-called “paper-clip” fold [20,21] that likely prevents aberrant intra- and intermolecular interactions that could lead to protein misfolding and aggregation. Whereas binding to MTs forces the tau MTBR into a semi-regular structure that can be resolved by cryo-electron microscopy (EM) [22], the N-terminal projection domain and the C-terminus remain largely unstructured/unresolved. Surprisingly, despite tau's likely importance for axonal MTs function and structure, *Mapt*^−/−^ mice are viable, and young animals show no overt developmental or cognitive phenotypes likely due to a compensatory upregulation of other MAP proteins [23]. However, even though rodents do not develop neurodegeneration *per se,* aged *Mapt*^−/−^ mice, with results varying between labs and mice strains, exhibit synapse loss and a range of cognitive defects that could be due to reported defects in synapses' long-term potentiation and long-term depression [14]. This raises the possibility that physiological tau might have direct and important roles in synaptic function.

### Post-translational modifications

Under normal physiological conditions, tau undergoes extensive reversible post-translational modifications (PTMs) that regulate its function [24], localization [25], and turnover [26]. Phosphorylation is the most extensively studied PTM [13], as one of the main regulatory mechanisms of tau function and MT binding, but hyperphosphorylation of specific amino acid residues is also closely associated with the aberrant accumulation and pathological inclusions of tau observed in post-mortem brain. Importantly, the levels of certain phosphorylated residues (e.g., pT271) have emerged as peripheral blood-based biomarkers [27]. Notably, several studies of AD detect tau hyperphosphorylation before amyloid aggregation [28]. Increased phosphorylation of tau inhibits its ability to bind to MTs [29,30], increasing the concentration of free tau and its aggregation propensity. In addition to phosphorylation, tau can also undergo acetylation, glycation, glycosylation, ubiquitination, sumoylation, nitration, methylation, and deamidation, the functional significance of which is the subject of ongoing research. Apart from acetylation and low levels of phosphorylation, which are also found in healthy brains, most of these PTMs have been specifically identified and studied in the context of disease (Fig. 1b) [31]. Another PTM observed in cell culture and animal models and to some extent in AD patient brains are tau truncations. These can have significant implications for the pathogenesis of tauopathies, including aggregation and spreading, as even truncations that do not directly affect the amyloid-forming core can significantly impact the structure of the tau fibrils formed [32].

### Tau in Alzheimer's disease

The first clinical description of AD that combined all major clinical symptoms with the post-mortem observation of Aβ plaques and NFTs was made by Alois Alzheimer in 1907 [33]. Only 80 years later, tau was identified as the constituent of the straight filaments (SF) and paired helical filaments (PHF) that make up NFTs, and the amyloid core of these tau inclusions was shown to be formed mainly by the MTBR domain of tau [[34], [35], [36], [37]]. Tau amyloid-type inclusions are not only observed in AD but also in a range of other neurodegenerative diseases termed tauopathies. Pathologies in which tau aggregation occurs as a secondary process relative to a primary insult, which in AD's case is Aβ aggregation, are classified as secondary tauopathies; while diseases in which tau aggregation is the main pathological process are classified as primary tauopathies [38]. This classification can be further stratified by which tau isoforms are primarily found in tau inclusions, 3R only, 4R only, or both 3R/4R, as is the case in AD [39]. Recent advances in cryo-electron microscopy have further uncovered that tau fibrils extracted from tauopathy patients have disease-specific tertiary and quaternary structures that are consistent across brains with the same type of disease pathology [40]. This tremendous advance has initiated a new ultrastructure-based classification of tauopathies.

Disease-associated tau differs in several other characteristics from its healthy protein counterpart. As mentioned earlier, pathological tau has several aberrant PTMs, the most well-studied being hyperphosphorylation [31]. Specific phosphorylation patterns have been shown to lead to the accumulation of tau in synapses and dendrites, where it exhibits a deleterious gain of function, disrupting neurotransmission and neuroplasticity [25,41]. Most notable, however, is the misfolding of hyper-phosphorylated tau into a β-sheet rich conformation, subsequent oligomerization, and growth into large amyloid fibrils through a polymerization-like reaction that incorporates native monomers at the end of the growing tau amyloid fiber (Fig. 1c). New filaments are generated by either breaking of mature fibrils into smaller fragments, often called seeds, or secondary nucleation on the fibril surface [42,43]. This is reminiscent of the misfolding events described for the prion protein (PrP^C^/PNRP) in diseases like Creutzfeldt-Jakob disease and scrapie [[44], [45], [46]], hence giving rise to the notion that misfolded tau has “prion-like” properties. Since tau is an intracellular protein, one might assume its aggregation only poses a direct risk to the initially “affected” neuron(s). However, a large body of literature supports the hypothesis that pathological tau's “prion-like” properties also include the ability to spread and induce aggregation of native tau in recipient cells [[47], [48], [49], [50]]. In support of this model, it has been shown that injection of human tauopathy brain homogenates into humanized wild-type tau mice can induce tau aggregation in the rodent brain [[51], [52], [53]]. The hypothesis that cell-to cell spreading of tau seeds is a driver of pathology is further supported by tau PET scans and histopathological studies that characterized the progressive spreading and accumulation of tau tangles over disease progression. Notably, in disease, the accumulation of misfolded tau starts in specific brain regions and spreads to interconnected areas in specific patterns [54,55]. How tau seeds transfer between donor and recipient cells is still the focus of current research, with several potentially parallel mechanisms proposed. Tau seeds can be released from neurons through unconventional secretion [56,57], through interaction with cell surface receptors like heparan sulfate proteoglycans [50], the lipoprotein receptor-related protein 1 (LRP1) [58], muscarinic receptors [59], and through small extracellular vesicles (EVs) named exosomes that can serve as vehicles for intracellular spread of tau seeds [49,60]. Evidence of pathogenic tau ‘hitchhiking’ inside EVs comes from cryo-electron microscopy studies showing the presence of small tau fibrils in EVs isolated from AD brains [61]. Despite the large body of literature supporting the prion-like behavior of tau, there are still many open questions, the main one being which part of the formation of tau fibrils is neurotoxic. Identification of the pathogenic forms of tau, which may change as the disease progresses, is critical to the successful development of effective tau-targeted therapeutics.

Likely a consequence of tau's many functions, its toxicity in disease is plausibly a result of combined loss of endogenous function and toxic gain of function by the hyperphosphorylated misfolded tau species. As mentioned above, tau knockout mice are viable and appear superficially healthy but exhibit some synapse loss and specific memory-related neurophysiological changes, indicating possibly understudied tau loss-of-function consequences in disease. A study focusing on the tau interactome in induced pluripotent stem cell (iPSC)-derived neurons, expressing either wild-type tau (control) or mutant tau associated with FTD (early-stage primary tauopathy model), showed significantly altered mutation-dependent interactions between tau and components of synaptic vesicles and mitochondria, affecting neuronal function and bioenergetics [62]. This is consistent with prior observations in an FTD patient iPSC-derived neuronal model with a different pathogenic tau mutation that exhibited mitochondrial hyperpolarization and increased oxidative stress [63]. As both studies represent early pre-aggregation onset disease models, tau loss of function likely contributes to neuronal dysfunction and death, as observed in AD and other tauopathies, but is unlikely responsible for all pathological changes. Regarding tau toxic gain of function, several tauopathy animal models show clear evidence that both overexpression of wild-type or disease-associated mutant tau are neurotoxic [64]. Mature tau fibrils and NFTs that can fill the whole soma of affected neurons, as observed with histopathological tau staining, while impressive, are also thought to be biologically relatively inert; potentially even providing some protection by sequestering smaller, highly interactive tau oligomers that are highly neurotoxic [[65], [66], [67], [68]]. This is supported by numerous studies that show pathological oligomeric tau-mediated dysregulation of core cellular pathways, including splicing, proteostasis, and neuronal function (e.g., glutamatergic signaling), as well as glia and microglia homeostasis, before neurofibrillary tangle formation [64,[69], [70], [71], [72], [73], [74]]. Therefore, targeting the *early* pathological form(s) of tau for removal is a highly promising therapeutic strategy for AD and other tauopathies.

### Pathological tau as a therapeutic target

Aβ plaques and tau NFTs are well-established co-pathologies in AD, but the exact extent to which specific tau proteoforms contribute to a loss of homeostasis and disease progression is still unknown. While Aβ aggregation is not a prerequisite for tau aggregation *per se*, as is known from primary tauopathies, amyloid plaques precede tau tangle formation in AD. Nevertheless, the rationale for targeting tau as a therapeutic approach comes from several lines of evidence that unequivocally demonstrate that tau actively contributes to disease both in AD and in heritable primary tauopathies. In genetic heritable forms of tauopathy, mutations in the *MAPT* gene lead to a spectrum of symptoms, some of which overlap with AD [8], where tau is the sole or main driver of neurodegenerative processes. Moreover, very recently presented clinical trial data regarding the approved *anti*-Aβ immunotherapies [75], suggests that gantenerumab can efficiently reduce amyloid plaques down to a level indistinguishable from a healthy person and slow, but not stop, AD progression if administered early, potentially because accumulation of tau tangles is not reversed and further buildup is only slowed down but not prevented [76,77]. Antibody-based therapeutics essentially sequester and mark the recognized antigen for degradation by microglia, the CNS immune cells. Involvement of the immune system in this manner has presented challenges, including undesired auto-immune reactions against vascular Aβ deposits that can result in micro- or even macro-hemorrhages [[78], [79], [80]]. While successfully targeting Aβ, targeting tau by passive immunotherapy faces additional barriers. Tau, as a constitutive intracellular protein, is shielded from the immune system. Even if free extracellular pathogenic tau is effectively sequestered, exosome-encased tau will likely remain unaffected and continue to spread tau seeds intercellularly. Several groups have independently demonstrated that reducing or clearing pathological tau species through various mechanisms can be beneficial in tauopathy model systems. One way this has been shown is through upregulation of the autophagy-lysosomal-pathway (ALP), a multi-component system of the proteostasis network that degrades and recycles lipids, small and large protein aggregates, and damaged organelles [81]. Reducing tau through increasing ALP activity rescues tau-mediated phenotypes in human and mouse models of tauopathy [[82], [83], [84], [85]]. This approach, however, does not specifically target native or misfolded tau but relies on tau becoming passively engulfed by the autophagic machinery that is likely to also degrade other proteins as “collateral damage.” Hence, increasing the effort to improve upon existing modalities to develop new targeted protein degradation (TPD) tools that act specifically on intracellular pathological tau is of critical importance for therapeutic development.

## Targeted Protein Degradation

The concept of TPD has existed since the early 2000s. It is an umbrella term for methods that direct a target protein of interest (POI) to one of the endogenous cellular degradation machineries. While TPD can utilize small molecules or biologics, a central design principle is their hetero-bifunctionality: one module (also called warhead) binds to the POI target for degradation, while the other module interacts with a component of the selected degradation pathway. The two modules are bound by a linker of variable length and chemical composition that provides the distance and flexibility optimized for the intended interaction. This sets TPD methods apart from antibody-based immunotherapies in that there is no recruitment of the immune system to remove pathological species and instead engage a specific protein degradation pathway. Of critical importance to the development of the field of small-molecule-based TPD is the growing in-depth understanding of the molecular mechanisms involved in protein degradation. While not representing the entirety of mechanisms, the main degradation pathways chosen for TPD and small molecule design are the ubiquitin-proteasome system (UPS), the ALP (and subcategories, e.g. macro-autophagy, chaperone-mediated autophagy), both for degradation of intracellular targets, and the endo-lysosomal system to target membrane proteins and extracellular targets.

**Fig. 2:**
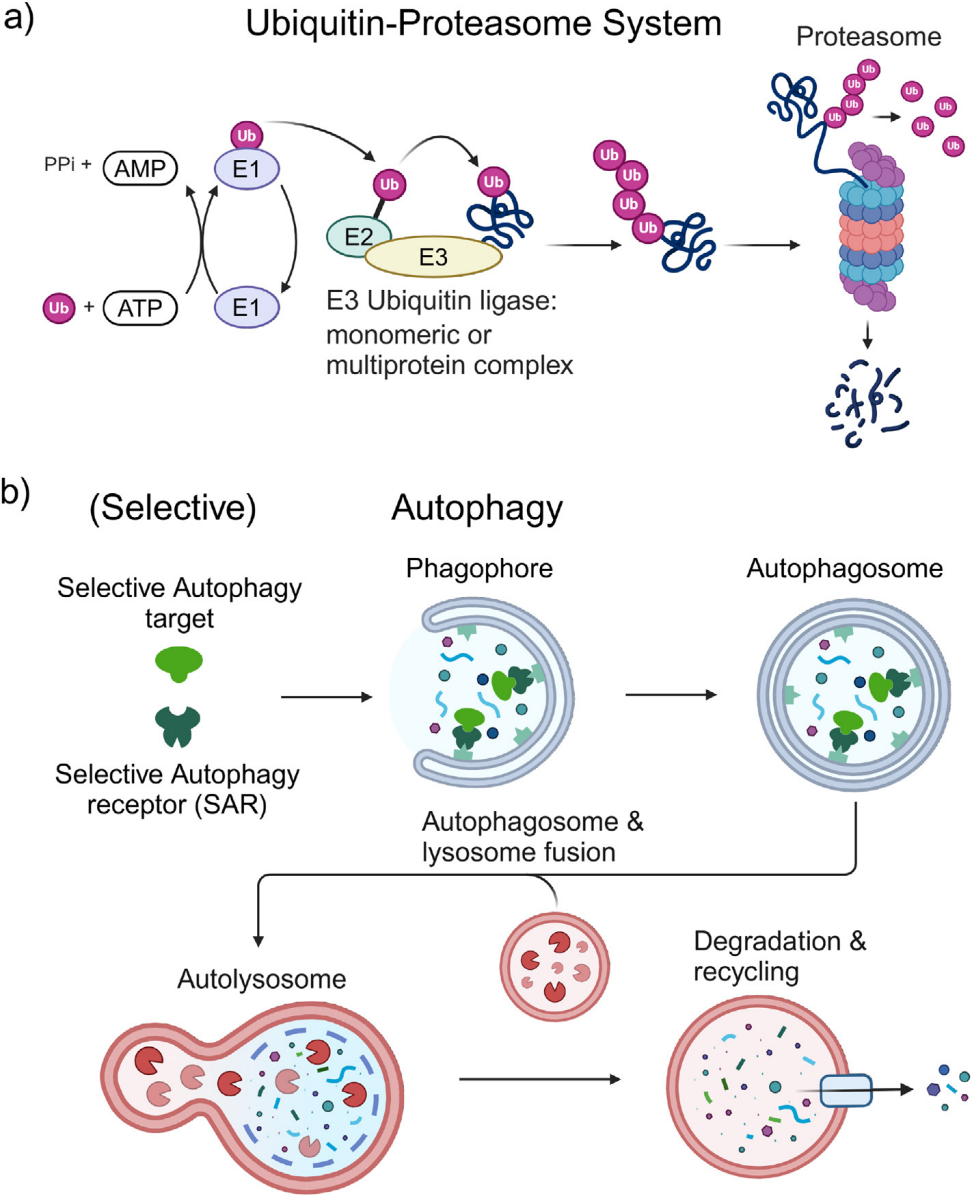
**Cellular degradation mechanismsutilized for targeted protein degradation strategies. (a)** The Ubiquitin-Proteasome-System marks proteins for degradation through the addition of K48 poly-ubiquitin chains. This is mediated by a cascade of E1 ubiquitin-activating enzymes, E2 ubiquitin-conjugating enzymes, and E3 ubiquitin ligases. Substrate specificity is provided by E3 ligases that can be monomeric enzymes or multiprotein complexes. Poly-ubiquitinated proteins are recognized either directly by the 19S regulatory particle of the proteasome or through the interaction of the proteasome with ubiquitin receptors. During translocation through the central pore of the 20S core particle, the substrate is simultaneously de-ubiquitinated, unfolded, and cleaved into small peptides. **(b)** Macroautophagy starts with the de novo formation of a lipid double membrane called a phagophore. The growing phagophore non-selectively encloses part of the cytosol but can also selectively sequester autophagy targets through the help of selective autophagy receptors (SARs) that bind substrates and proteins localized on the inside of the growing phagophore. Once the phagophore is closed, it is called an autophagosome. Fusion of the autophagosome with a lysosome leads to degradation of the inner autophagic membrane and its contents by lysosomal hydrolases. Lastly degradation products are exported out of the autolysosome for recycling. Created in BioRender. Sandhof, C. (2024) BioRender.com/g60h412.

The UPS acts in a stepwise manner, using an E1-E2-E3 enzyme cascade reactions to covalently attach ubiquitin to lysine side chains of target proteins for degradation by the 26S proteasome system (Fig. 2a) [86]. The ubiquitin degron tag on the protein is recognized by the regulatory particle (19S) of the proteasome, which channels the now unfolded polypeptide through the central pore of the proteasome (20S core particle) where the polypeptide chain is cleaved into smaller peptides [86]. The central pore of the proteasome is small and can only process single proteins that are either already unfolded or easily unfolded through the pulling forces exerted by the proteasome during degradation [87], constituting one of the main bottlenecks for the degradation of protein aggregates. Substrate specificity for the UPS is provided by the E3 ubiquitin ligases and the attachment of the ubiquitin degron tag to a protein to be degraded, with emerging opportunities to achieve cell-type or tissue-selective pharmacology based upon the coincident of expression of specific E3 ligase subunits that exhibit restricted expression [88].

While there are sub-classes of autophagy that target single proteins for degradation, like chaperone-mediated autophagy (CMA) [89], the most well-studied part of the ALP is macroautophagy (from here on just “autophagy”), which can engage and degrade large proteins, protein complexes, and protein aggregates [86]. This is achieved by the de novo formation of a lipid double membrane (termed phagophore) initiated by activating the ULK1 or ULK2 complex [90]. The growing phagophore can semi-randomly engulf part of the cytosol in bulk (for nutrient recycling) or selectively engulf proteins, forming an autophagosome that subsequently fuses with a lysosome (autolysosome) for proteolytic degradation of its contents (Fig. 2b). Bulk autophagy provides the cell with the energy and basic molecular building blocks to keep functioning. Selective autophagy is mediated by specialized selective autophagy receptors (SARs; e.g. p62, a.k.a. sequestosome/SQSTM1), which bind to the protein cargo and funnel it to degradation by interacting with parts of the autophagy machinery that localize to the inner membrane of the growing phagophore (Fig. 2b) [91]. In the case of organelles or large immobile protein aggregates, phagophore formation is induced locally via recruitment of the autophagy initiation complexes by clustering of SARs on the autophagy substrate. This provides autophagy-based TPD with several potential avenues to direct POIs for degradation by either tethering them to SARs or by recruiting the POI to the inner membrane of the developing phagophore in a SAR-like fashion.

The TPD field could broaden the potential for treatment of a range of diseases that have known molecular drivers, like mutant oncogenic proteins in cancer and aggregating proteins in neurodegeneration, that were once thought to be undruggable but can now be potentially targeted by engineering specific TPD molecules. Since 2001, the expansion of TPD strategies has led to a boom in preclinically-tested small molecules. In oncology, the field driving TPD research, two novel therapeutics have already advanced to Phase II and III clinical trials [92,93]. In neurology, the initial success with Aβ-targeting antibodies for AD may spur the field to advance TPD therapeutics for other neurodegeneration targets such as tau and α-synuclein (α-Syn), the major amyloid aggregate forming protein in synucleinopathies like Parkinson's disease, among others. While no TPD molecule has yet entered clinical trials for neurodegenerative diseases, there have been several TPD methods directed against tau that demonstrate efficient degradation in various model systems (Table 1), which we aim to highlight here.

**Table 1:** Lead TAU TPD modalities developed by academic groups.

Name	TPD modality	Tau warhead	Linker	Degradation Module	Tested in	Ref.
TH006	Peptide PROTAC	YQQYQDATADEQG	GSGS	ALAPYIP Binds VHL	• Heterologous cell model (N2a) • *ex vivo* neurons and *in vivo* (3xTg-AD mice)	[95]
Peptide 1	Peptide PROTAC	YQQYQDATADEQG	GSGS	LDPETGEYL Binds Keap1	• Heterologous cell models (N2a, SH-SY5Y, PC12)	[96]
QC-01–175	Small molecule PROTAC	caiq3-74 (T807 derivative)	[PEG]_2_	Pomalidomide derivative Binds CRL4^CRBN^	• Patient iPSC derived *ex vivo* neurons	[101]
C8	Small molecule PROTAC	Quinoxaline derivative	[Alkyl]_8_	Thalidomide Binds CRL4^CRBN^	• Heterologous cell models (HEK293T-hTau) • *In vivo* (C57BL/6 mice ​+ ​Tau AAV)	[110]
C004019	Small molecule PROTAC	Tau Binder ID220149	[PEG]_3_	VHL binder	• Heterologous cell models (HEK293T-hTau, SH-SY5Y) • *In vivo* (C57BL/6, 3xTg-AD mice)	[105]
Compound I3	Small molecule PROTAC	THK5105 derivative	Triazole-[PEG]_3_	Thalidomide Binds CRL4^CRBN^	• Heterologous cell models (PC12) • *In vivo* (ICR mice)	[107]
T3	Small molecule PROTAC	thioflavin-T analogue	[PEG]_3_	Pomalidomide Binds CRL4^CRBN^	• Heterologous cell models (HEK293T, SH-SY5Y) • *In vivo* (C57BL/6 mice; SD rats)	[113]
HyT-Tau-CPP	Hydrophobic tagging	YQQYQDATADEQG	none	Adamantane	• Heterologous cell models (N2a) • *In vivo* (B6129SF2/J ​+ ​3xTg-AD mice)	[119]
D14	*D*-Peptide DEPTAC	YQQYQDATADEQG	[PEG]_6_	RTPPKSP Binds PP2A-Bα	• *Ex vivo* SD rat primary neurons • *In vivo* (C57BL/6, 3xTg-AD, hTau368 mice)	[123]
Tau2-8	Small molecule-peptide hybrid DEPTAC	PI-2620	[PEG]_8_	AP1867 Binds FKBP12 tags	• Heterologous cell models (HeLa)	[127]
D20	Peptide DEPTAC	DVWMINKKRK	[PEG]6	PRVRF Binds PP1	• Heterologous cell model (HEK293T, N2a) • *ex vivo* neurons (rat primary neurons) • *in vivo* (3xTg-AD and P301L mice)	[124]
PBA-1105	AUTOTAC	PBA	[PEG]_2_	YOK-2204 p62 ligand	• Heterologous cell model (SH-SY5Y-tauP301L cells)	[141]
Anle138b-F105	AUTOTAC	Anle138b	[PEG]_3_	YT-8-8 p62 ligand	• Heterologous cell model (SH-SY5Y-tauP301L cells) • *In vivo* (TauP301L-BiFC mice)	[141]
R–Nb	RING-nanobody degrader	F8-2 (S54L ​+ ​T127A) nanobody	GGGGS	TRIM21 RING domain	• Heterologous cell model (seeded HEK293-tauP301S-venus cells) • *Ex vivo* neurons and *in vivo* (Tg2541 mice)	[155]
Tau-RING	RING-bait degrader	Tau		TRIM21 RING domain	• Heterologous cell model (recombinant tau seeded HEK293-tauP301S-venus cells, AD or PSP brain derived tau seeded HEK293-0N3Rtau and HEK293-0N4Rtau cells, BHK– P301S tau-venus cells) • *Ex vivo* neurons and *in vivo* (Tg2541 mice)	[157]

### TAU TPD approaches

#### PROTACs

**Fig. 3:**
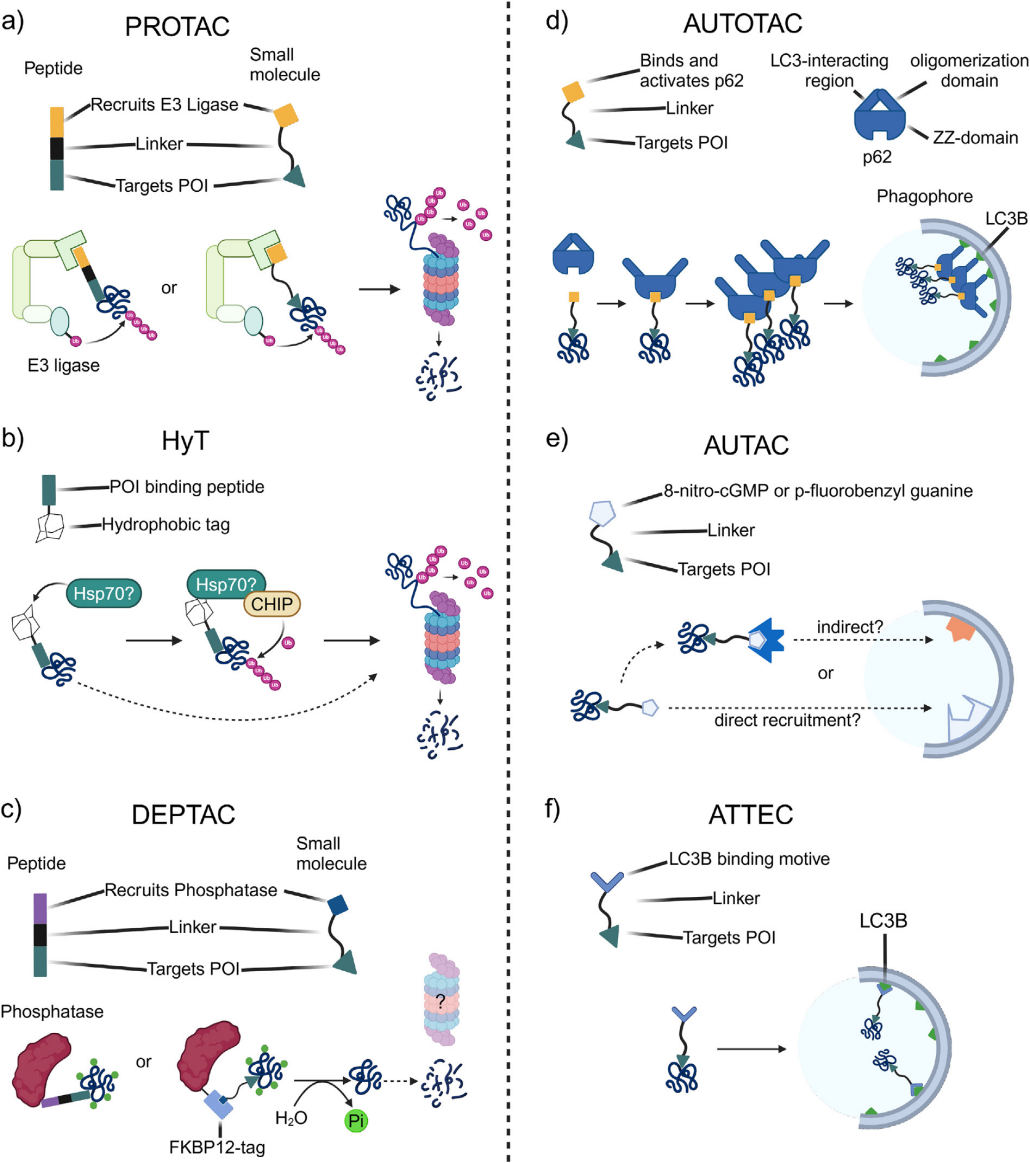
**Peptidic and small molecule TPD methods. (a)** Tau PROTACs are either peptide or small molecule-based, with one module (also called warhead) binding the protein of interest (e.g., tau) and the other binding to an E3 ligase subunit protein. Peptidic tau PROTACs bind to the microtubule-binding repeats of tau, while small molecule tau warheads, which are derived from amyloid binding molecules, preferentially recognize structural motives present in tau oligomers. The proximity between the E3 ligase and tau leads to poly-ubiquitination of the latter and subsequent proteasomal degradation. **(b)** Hydrophobic tagging-based TPD agents bind tau through a peptide sequence and recruit molecular chaperones (potentially HSP70) to a strongly hydrophobic small molecule moiety. Interaction of the chaperone with the E3 ligase CHIP poly-ubiquitinates tau and leads to its proteasomal degradation. **(c)** DEPTACs facilitate proximity between tau and phosphatases, leading to the dephosphorylation of tau. Aberrant phosphorylation of tau prevents its degradation enabling DEPTACs to indirectly lead to degradation of accumulated tau. Currently published small molecule-based DEPTACs (PhosTACs) rely on engineered phosphatases with defined tags due to the unavailability of well-understood phosphatase recruiting small molecule moieties. **(d)** AUTOTACs not only form a tau-p62 tertiary complex but also induce a structural change in the selective autophagy receptor p62 by binding to its ZZ-domain. This induces p62 oligomerization, resulting in the induction of autophagy and sequestration of tau into the autophagy lysosomal pathway. **(e)** AUTACs utilize 8-nitro-cGMP or the chemically similar *p*-fluorobenzyl guanine to direct warhead-bound POIs toward autophagic degradation. It is still unclear if this is facilitated indirectly by dedicated selective autophagy receptors or if the 8-nitro-cGMP binds to a protein on the inside of a growing phagophore. **(f)** ATTECs combine LC3B, an integral part of the autophagic machinery localized at the phagophore membrane, binding motives with POI binding warheads. Created in BioRender. Sandhof, C. (2024) BioRender.com/y18n942.

**Fig. 4:**
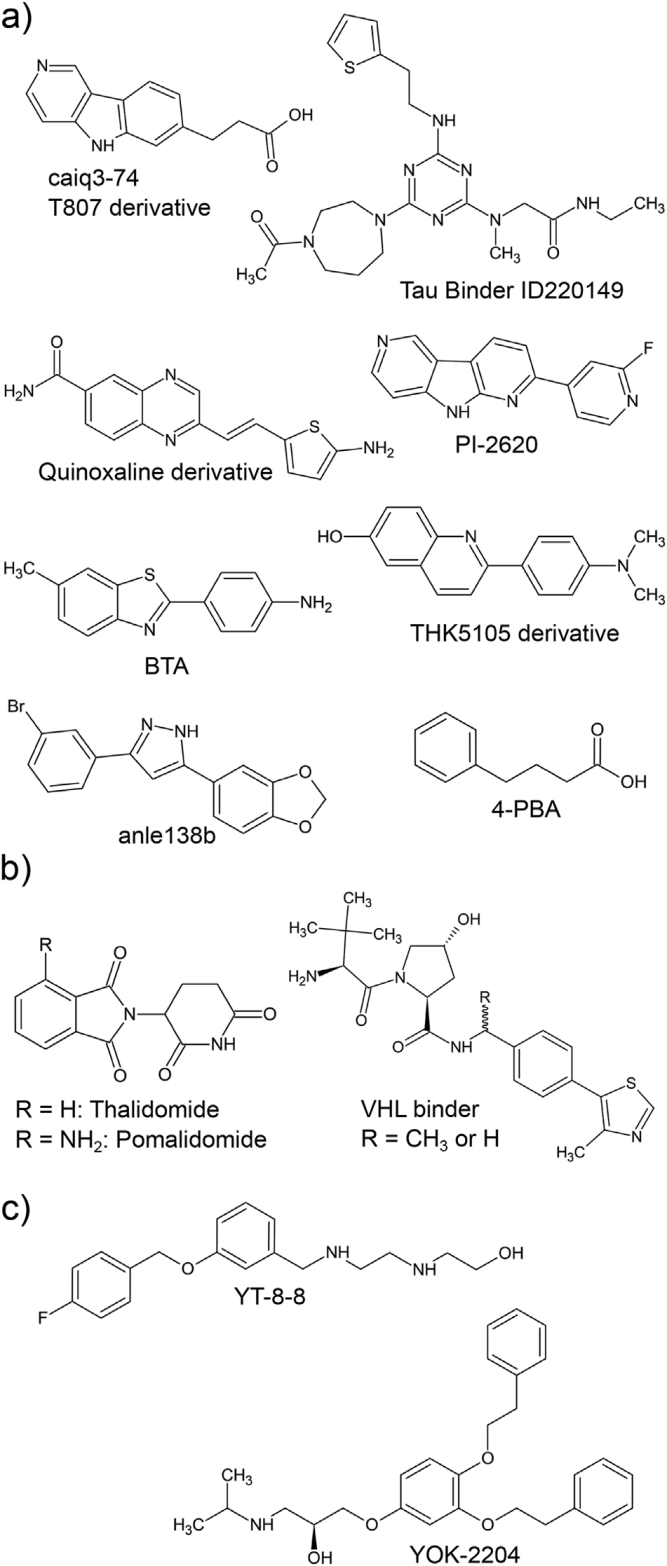
**Chemical structures used as modules for the design of lead tau targeting TPDs. (a)** Tau warheads. **(b)** E3 ligase recruiting modules used in tau PROTACs. **(c)** Adamantane is the only tag so far utilized in tau HyT design. **(d)** p62 ZZ-domain binders used for the design of tau targeting AUTOTACs. Created in BioRender. Sandhof, C. (2024) BioRender.com/r47z297.

The first PROteolysis Targeting Chimera (PROTAC) was a bifunctional small molecule and peptide hybrid that linked a peptide sequence recognized by the SCF (Skp, Cullin, F-box) E3-ligase complex with a small molecule inhibitor of an aminopeptidase (POI). Upon ternary complex formation via the PROTAC, the aminopeptidase was brought into proximity of the SCF E3-complex for ubiquitination and subsequent proteasomal degradation [94]. Unsurprisingly, the first tau-targeting PROTACs were also peptide-based (Fig. 3a) and recruited either the SCF-E3, the von Hippel-Lindau (VHL) Cullin RING E3 ligase [95], or the Keap1-Cul3 ubiquitin E3 ligase [96,97] to ubiquitinate tau. In these studies, tau binding specificity was achieved using a tubulin-derived peptide. As a result, this approach is likely only able to degrade monomeric tau, i.e., the MT-binding competent tau, since misfolded and aggregating tau is unlikely to bind to the tubulin-derived peptide. Advances in chemical biology provided the tools for developing the next generation, small molecule-based PROTACs [98], the most developed and advanced type of PROTACs in terms of clinical studies. Their basic building blocks are: a small molecule warhead that binds to the POI, a variable linker, and an E3 ligase binder. Importantly, the warhead itself does not need to change target activity but only must bind to it with desirable pharmacokinetics. This design is favorable over peptide-based approaches as it increases molecular stability and cell permeability (which is particularly important for targets in the CNS), and benefits from access to a large chemical space for the building blocks of each PROTAC module and the target POIs. This strategy now affords the possibility to target CNS diseases and proteins that until recently remained refractory to conventional drug development efforts. The development of tau-targeting PROTACs benefited greatly from advances in the field of tauopathy biomarkers and diagnostics that spurred the development of several PET tracers [99] that bind preferentially to misfolded and amyloid tau species in living patient brains [100]. Particularly, T807 (flortaucipir) is the most advanced and clinically used tau-ligand PET tracer in neurology. The first small molecule-based tau PROTAC (QC-01-175) with evidence for efficacy in tauopathy patient iPSC-derived neuronal-based assays was developed in our group by optimally linking a minimal tau binding motive derived from T807 (Fig. 4a) and the small molecule pomalidomide (Fig. 4b), which recruits the CRL4^CRBN^ E3 ligase complex (Fig. 3a) [101]. The QC-01-175 PROTAC represents a large specificity improvement over previous peptide-based tau PROTACs. It was the first small molecule shown to be able to selectively degrade disease-associated mutant tau (A152T and P301L) in tauopathy patient iPSC-derived neuronal cell models in a manner that could be blocked by a neddylation inhibitor (MLN-4924), demonstrating dependency on the E3 ligase activity of CRL4, as well as by an inhibitor of the major protease activities of the proteasome (carfilzomib). Additional mechanistic controls performed included demonstrating that the effects of QC-01-175 on tau degradation were blocked by cotreatment with excess T807 and lenalidomide, which prevent proper ternary complex formation. QC-01-175 was also shown to lack an appreciable impact on levels of wild-type tau from healthy control neurons or neuronal viability. Critically, this selective degradation of tau only in the context of neurons from affected patients and not healthy controls provides an informative example of leveraging patient-specific iPSC models to counter-screen early in the discovery process against undesired off-target effects that can confound mechanistic interpretation (e.g., effects on general cell viability or processes like transcription and translation of the *MAPT* gene). Moreover, degradation of mutant tau with QC-01-175 rescued mutant tau-dependent stress vulnerability in the patient iPSC-derived neurons, revealing a beneficial impact of removing tau on enhancing resiliency toward cellular stressors. Interestingly, this PROTAC also mitigated mitochondrial fragmentation and oxidative stress when subsequently tested in SH-SY5Y cells treated with synaptic fractions from postmortem amyotrophic lateral sclerosis (ALS) patient brains that are enriched in mis-localized hyper-phosphorylated tau [102], revealing the potential of PROTACs in a tau co-pathology context. These observations add weight to the hypothesis that tau degradation might benefit a range of neurodegenerative diseases with tau pathology. Subsequently, in addition to expanding the structure-activity-relationship around QC-01-175 and demonstration of efficacy with VHL-based degraders in tauopathy-patient iPSC neuronal models for the first time [103], as well as a diverse range of small molecules claimed in patent applications [104], several other groups have reported in the academic literature PROTACs that efficiently degrade tau. C004019 [105] combines a VHL E3-ligase binding module (Fig. 4b) with a tau ligand that was originally identified in a chemical screen for 2N4R tau monomer binding compounds that also inhibit tau aggregation [106]. C004019 was shown to degrade tau in a mouse model expressing human wild-type tau, reduced tau levels and rescued cognitive and synaptic phenotypes in the 3xTg AD mice, providing the first proof for *in vivo* efficacy [105]. In 2022, Liang et al. developed the PROTAC I3 [107] with a thalidomide-derived CRL4^CRBN^ binding module (Fig. 4b) and a tau ligand derived from the pre-clinical tau PET tracer THK5105 (Fig. 4a) [108]. Treatment with compound I3 for 24h at 25 ​μM and higher concentrations reduced endogenous tau in PC12 ​cells and protected against Aβ-peptide induced toxicity. In PC12 ​cells overexpressing tau, treatment with compound I3 rescued mitochondrial localization defects. Another tau PROTAC named C8, similar to QC-01-175, used thalidomide as the CRL4^CRBN^ binding module and a quinoxaline (Fig. 4b) derivative [109] as the tau binding module [110]. C8 was shown to slightly reduce hyperphosphorylated tau in an AD mouse model [110]. Additional studies have now expanded the structural diversity of tau PET ligand-based warheads incorporated into PROTACs with evidence for efficacy in HEK293 ​cells as well as *in vivo* in the rTg4510 AD mouse model [111,112]. Recently, a tau and α-synuclein dual targeting PROTAC named compound T3 was described [113]. This PROTAC combines pomalidomide (Fig. 4b) as the CRL4^CRBN^ recruiting module with 2-[49-(methylamino)phenyl]-6-methylbenzothiazole (BTA; Fig. 4a), a derivative of the amyloid fiber binding thioflavin-T, for binding to misfolded α-synuclein and tau. While no data on cognitive function was presented, the T3 PROTAC efficiently reduced pathological α-synuclein and tau in a chemically induced Parkinson's disease mouse model [113].

#### Hydrophobic tagging (HyT) degraders

HyT degraders could be considered a subcategory of PROTACs as they also rely on proteasome-mediated degradation. However, the term PROTAC has been exclusively used for molecules that directly facilitate proximity between a POI and an E3 ligase via ternary complex formation. As the name suggests, instead of an E3 ligase binding module, HyTs have a strong hydrophobic module [114]. This mimics the exposure of hydrophobic amino acid chains by unfolded proteins that are recognized by molecular chaperones [115] that, in turn, facilitate the refolding of these proteins or direct them to proteasomal degradation [116] through the recruitment of the E3 ligase CHIP (Fig. 3b) [117]. While HyTs that recruit chaperones to a target POI can promote protein ubiquitination, there are exceptions with at least one HyT shown to directly interact with the proteasome and induce protein degradation circumventing chaperone-CHIP-mediated POI ubiquitination [118]. So far, only one tau-targeting peptide-based HyT has been described. This HyT combines an adamantane hydrophobic module, a tau-binding peptide, and a cell-penetrating peptide (CPP) motif (HyT-Tau-CPP) [119]. While HyT-Tau-CPP led to some tau degradation in N2a cells overexpressing Tau-eGFP, treatment at a concentration of 50 ​μM for 24h only resulted in a 20 ​% tau reduction, suggesting limited therapeutic applicability given challenges in achieving sufficient and sustained brain exposure.

#### DEPTACs and PhosTACs

DEPhosphorylation TArgeting Chimeras (DEPTACs) represent a novel strategy for modulating protein phosphorylation and are being tested for removing disease-associated hyper-phosphorylated tau. In 2021 Zheng et al. [120], reported the first DEPTAC targeting tau for dephosphorylation by harnessing the function of one of the main tau phosphatase enzymes, protein phosphatase 2A (PP2A) [121]. The original DEPTAC was based on a tau binding peptide, a short linker, a PP2A-Bα phosphatase recruiting peptide, and a CPP (Fig. 3c). Tested in rat primary neurons overexpressing human tau, the DEPTAC efficiently reduced tau phosphorylation at several disease-associated amino acid residues but also decreased the levels of total tau. In an inducible tau_1-368_ fragment overexpression mouse model (hTau368) [122], DEPTAC treatment reduced phosphorylated and total tau, increased spine density, and improved cognitive impairment. The same group subsequently published an improved version (D14) of their initial DEPTAC [123], where the GSGS-linker was replaced with a PEG-linker, and the PP2A-Bα recruiting peptide was shortened to reduce the DEPTAC's molecular weight. Additionally, they synthesized the peptidic part of D14 from *D*-type amino acids to circumvent degradation by peptidases. Like the parental tau DEPTAC, D14 administration reduced tau phosphorylation and total tau in rat primary neuronal cultures, intracerebral hTau_1-368_-AAV injected C57BL/6 mice, 3xTg-AD mice, and hTau368 mice, but at much lower concentrations. Intravenous injection of D14 furthermore rescued tau-dependent neuroplasticity and cognitive phenotypes in the hTau368 mouse model. In a more recent optimization, this group identified a combination (D20) of a protein phosphatase 1 (PP1) recruiting peptide, a PEG6 linker, a tau aggregation inhibiting peptide, and a cell-penetrating peptide as the most potent DEPTAC [124,125]. D20 was able to significantly reduce tau phosphorylation in human tau overexpressing rat primary neuron cultures at 10 ​μM. Intravenous injections in P301L and 3xTg AD mice recapitulated the *ex vivo* observations, reducing phosphorylated tau and total tau, as observed with other tau DEPTACs. Moreover, D20 injections in 3xTg AD mice rescued cellular disease-relevant phenotypes, like microtubule bundle length, mitochondria numbers, and synapse density, as well as memory impairments observed in behavioral tests [124].

Shortly after the first tau DEPTAC was published, Crews and colleagues developed a small molecule version of the DEPTAC that they named “PhosTAC” [126]. Their proof-of-concept study relied on heterologous co-expression of an FKBP12-tagged phosphatase and a Halo-tagged POI that were brought into proximity by a PhosTAC containing an FKBP12 ligand (AP1867) and a chloroalkane group with high affinity for the HaloTag. Building on this approach, the same group later published another study, this time specifically targeting tau for dephosphorylation [127]. In this study, the authors first applied their proof-of-concept approach to tau in a cellular model with inducible overexpression of a HaloTag-tau construct (POI) and transient FKBP12-tag-PP2Aα expression. Treatment with a PhosTAC (“PhosTAC7”) that induces the formation of a ternary complex between these two constructs by binding to the HaloTag and FKBP12, led to rapid dephosphorylation of tau at two sites (T181+T231) by 75 ​%, and a ∼30 ​% decrease in total tau when treatment time was extended to three days. This decrease was dependent on proteasomal activity, as shown by co-treatment with the proteasomal inhibitor MG132 that abolished the reduction in total tau. The authors subsequently replaced the HaloTag warhead with PI-2620 (Fig. 4a), a next-generation, flortaucipir-derived tau PET tracer for direct recognition of tau (Fig. 3c) [128]. Treatment with this new PhosTAC compound (“Tau2-8”) led to a maximal reduction in phosphorylation by 50 ​% though no longer decreased total tau, probably due to a lower dephosphorylation efficiency [127]. Hence, while not specifically designed to reduce protein levels, tau dephosphorylation by DEPTACs leads to proteasome-dependent degradation of tau in a disease model in line with reports that specific tau phosphorylation sites inhibit its degradation [129]. For this reason, phosphorylation-targeting-chimeras can also be considered under the umbrella of TPD [130].

#### AUTOTACs

Sequestesome-1 (p62/SQSTM1) has many cellular functions but is most well-known to act as a SAR [[131], [132], [133]]. In autophagy, p62 recognizes ubiquitinated substrates (especially K63-poly-ubiquitin) through its ubiquitin-associated (UBA) domain, forms p62 inclusion bodies in a PB1 domain-dependent manner, and binds to microtubule-associated proteins 1A/1B light chain 3B (MAP1LC3B, i.e., LC3B) located at the phagophore membrane via its LC3-interacting region (LIR) motif [131,[134], [135], [136], [137]]. In 2017, Cha-Molstad et al. [138] discovered that p62 can serve as a recognin for N-end rule pathway proteins in the cell when the UPS is perturbed. Under proteasomal inhibition, N-terminal arginylated proteins preferentially bind to the ZZ domain of p62 rather than proteasome receptors [138,139]. Substrate binding to p62 changes its conformation and releases it from auto-inhibition. The resulting exposure of its PB1 domain facilitates self-oligomerization and recruitment of substrates to the phagophore as well as induction of autophagy, eventually resulting in N-terminal arginylated cargo degradation via the ALP [138,139]. To utilize this endogenous mechanism of p62-mediated autophagy activation, these researchers developed small molecules that mimic N-terminal arginylation and allosterically bind the p62-ZZ domain [138,140]. Treating a Huntington's disease-based HeLa cell model with the ligands resulted in the activation of autophagy and clearance of mutant huntingtin proteins, showing potential therapeutic relevance of the methodology for neurodegenerative diseases [138].

To target other POIs for degradation, groups led by Yong T. Kwon and Bo Y. Kim went on to develop bifunctional degraders with p62-ZZ ligands (Fig. 4c), bound to ligands for several different POIs, thereby creating the AUTOphagy-TArgeting Chimera (AUTOTAC) platform [141]. As a proof-of-concept, they showed effective degradation of numerous disease-relevant substrates, among them tau, via the same p62-self-oligomerization mechanism as the original p62-ZZ ligands (Fig. 3d) [141]. These tau-targeted AUTOTACs showed effective tau clearance with increased doses when p62-ZZ ligands were linked to either 4-phenylbutric acid (PBA) or Anle138b as the tau warhead (Fig. 4a). PBA is a U.S. Food and Drug Administration approved drug (outside of neurological indications) that functions as a chemical chaperone by binding to exposed hydrophobic regions of proteins to prevent aggregation [142], whereas Anle138b has been shown to bind neurotoxic oligomers of PrP, α-Syn, and tau [143,144] and is currently in clinical trials for Parkinson's disease [145]. Interestingly, the compound utilizing PBA seemed to show a hook effect, whereas the compound utilizing Anle138b did not [141]. Otherwise, both showed ALP-mediated clearance of total and insoluble tau species in a P301L-tau SH-SY5Y cell model, and the PBA-AUTOTAC additionally showed effective clearance of total, phosphorylated, and insoluble tau in a humanized-P301L-tau mouse model [141]. Since then, AUTOTACs have been further developed to specifically target α-synuclein in Parkinson's disease models, confirming the p62-oligomerization mechanisms *in cellulo* and exploring PK/PD properties *in vivo* [146]. Furthermore, the lead AUTOTAC from this study, ATC161, has recently been approved as an *Investigational New Drug* by the Korean Food and Drug Administration [147], showing the therapeutic potential of using AUTOTACs to target a range of proteins in neurodegenerative disease.

#### TRIM-away and TRIM21-derived TPDs

**Fig. 5:**
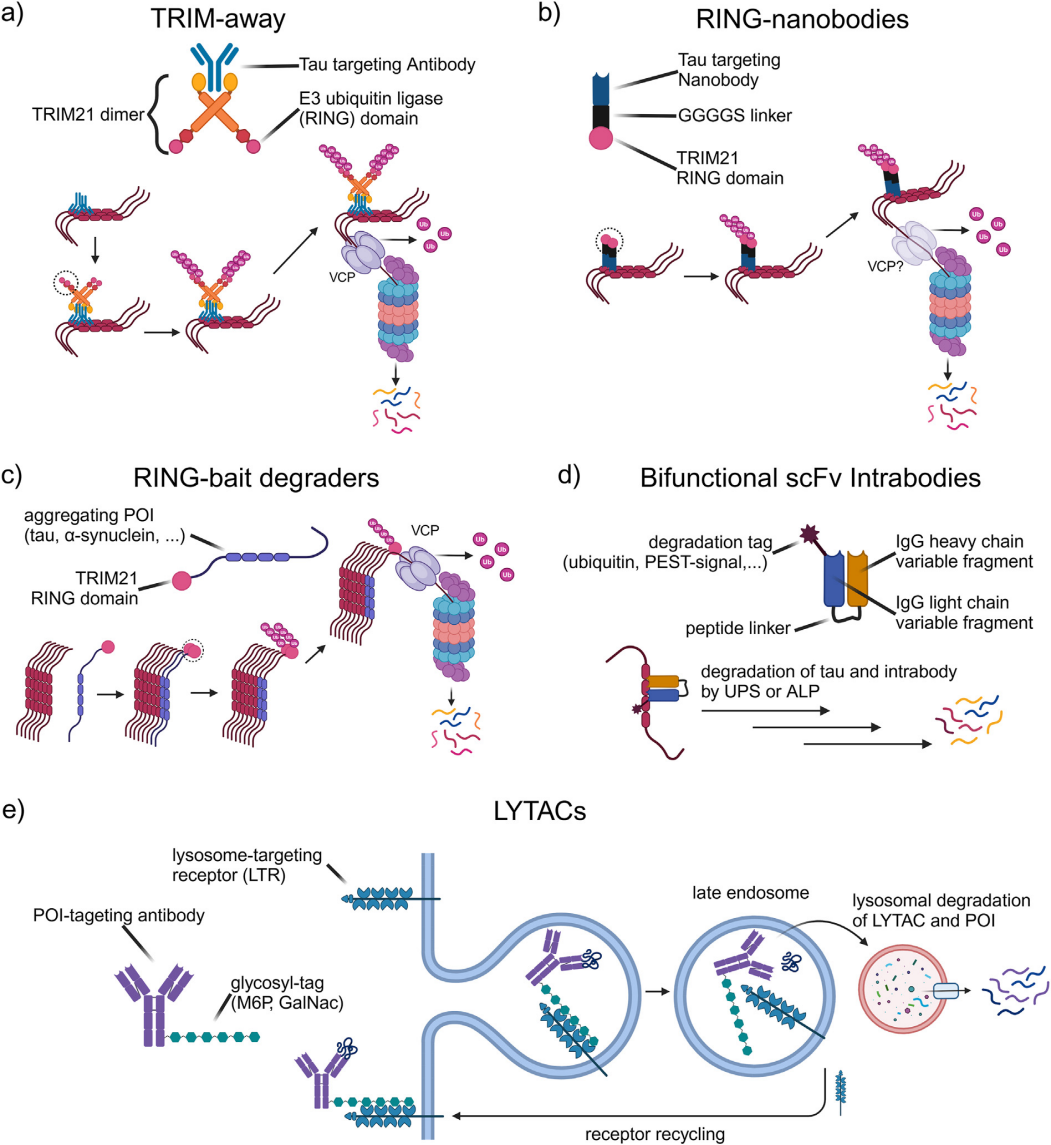
**Antibody-derived TPD methods. (a)** TRIM-away utilizes the intracellular antibody receptor TRIM21. Antibody clustering on tau oligomers leads to proximity-induced TRIM21 E3 ligase domain oligomerization and auto-ubiquitination, while bound monomers are unaffected since TRIM21 E3 domain homo-dimerization is required for ubiquitination activity. The TRIM-21-antibody-tau complex is then unfolded by valosin-containing protein (VCP) and degraded by the proteasome. **(b)** The RING domain-nanobody (R–Nb) approach represents a miniaturized version of the TRIM-away technique. Tau degradation is achieved through nanobodies fused to the RING E3 ligase domain of TRIM21 and acts in a similar manner as TRIM21-mediated degradation. Involvement of VCP for unfolding before proteasomal degradation is hypothesized but has not been shown yet. **(c)** RING-baits are fusion constructs of POIs that aggregate through templated misfolding and the TRIM21 RING domain. Incorporation of the RING-bait into the growing aggregate leads to RING-RING domain interactions, poly-ubiquitination, unfolding by VCP, and proteasomal degradation of the whole aggregate. **(d)** Bifunctional scFv intrabodies are minimal versions of IgG antibodies consisting of only the antigen-binding variable fragments fused together with a peptide linker. Degradation of the intrabody-tau complexes is facilitated by the addition of degradation tags to the scFv. The choice of the tag decides if the complex is degraded through ubiquitin-dependent or independent proteasomal degradation or autophagy. (e) LYTACs bind their target via their antibody component, and LTRs through an LTR-specific glycosyl tag. Activation of the LTR by the tag leads to internalization of the LYTAC-POI complex, dissociation from the LTR in late endosomes, and lysosomal degradation of the LYTAC and the POI while the LTR is recycled back to the cell surface.

TRIM-away is an antibody-based TPD method that targets intracellular proteins for degradation through the endogenous Tripartite motif containing-21 (TRIM21) pathway, bridging immunotherapy and TPD. TRIM21 is an intracellular, homodimeric antibody receptor with a high affinity for the Fc region of antibodies. It mediates an intracellular defense mechanism against antibody-bound pathogens that manage to enter the cellular cytosol [148,149]. This mechanism has been termed antibody-dependent intracellular neutralization (ADIN). TRIM21 is also an E3 ligase that is activated upon clustering of several antibodies bound to the same target, each of which gets recognized by a TRIM21 dimer, leading to auto-poly-ubiquitination (Fig. 5a) [150]. This targets the antigen-antibody-TRIM21 complex for unfolding by the AAA+ ​ATPase valosin-containing protein (VCP) and subsequent proteasomal degradation [151]. The first study utilizing the TRIM21-proteasome degradation pathway for TPD was published in 2017 by Clift et al. [152]. This study showed that microinjection or electroporation of antibodies into cells leads to rapid degradation of antibody-targeted POIs in a TRIM21-dependent manner. Subsequent studies demonstrated that antibody-bound tau seeds lose their ability to seed the aggregation of native cytosolic tau because they were rapidly degraded through ADIN [153]. Moreover, tau-targeting immunotherapies in mouse AD models were shown to remove tau also mainly through ADIN and not through antibody-mediated tau uptake and microglia degradation, as proposed [154]. Of note, the latter study also demonstrated that insoluble oligomeric tau is more susceptible to ADIN likely because these tau species are bound by more than one antibody, which aligns with the observation that TRIM21 activation necessitates clustering on the target.

A method that is taking this approach one step further combines tau-specific nanobodies with the E3 RING ligase domain of TRIM21 (R–Nb) that has recently been published by McEwan and colleagues (Fig. 5b) [155,156]. Transient expression of R-Nbs with an affinity to tau below 211 ​nM led to the degradation of aggregated but not soluble tau in HEK T-REx cells constitutively expressing venus-tagged tau-P301S (TV cells). Stereotaxic injection of mice expressing human tau-P301S with an adeno-associated virus (AAV) vector-based R–Nb construct decreased phosphorylated and aggregated insoluble tau levels in the injected hemisphere but did not affect total tau levels. Using a CNS-targeting AAV expression vector and intravenous injection (IV), the previous brain-localized observations were replicated at a wider CNS level. Surprisingly, preliminary data from the McEwan lab showed that R–Nb AAV IV injected tau-P301S mice had to be culled earlier than littermates injected with a control construct [156]. While the difference was not statistically significant, it demonstrated that this approach is still in its early stages and more in-depth *in vivo* treatment protocols are needed to demonstrate the potential future therapeutic applicability of R-Nbs for tauopathies.

The most recent TRIM-away derived TPD approach is RING-bait degraders [157]. The first, and as of the compilation of this review, only RING-bait degrader is a C-terminal fusion of the TRIM21 RING domain to 0N4R P301S-tau (P301S tau-RING). Conceptually, RING-bait degraders hijack the templated misfolding process of amyloid aggregating proteins, like tau, by integrating into the growing aggregate (Fig. 5c). This leads to RING-RING domain interaction, poly-ubiquitination, VCP mediated unfolding [157], and proteasomal degradation of the whole aggregate. Lentiviral expression of tau-RING in TV cells prevented tau seed-mediated aggregation in >95 ​% of the cells over 72h. Conversely, tau-RING expression in TV-cells, that were seeded beforehand and stably propagate tau-venus aggregates (TVA cells), cleared ∼80 ​% of aggregated tau over 72h compared to control transfected TVA cells. In both situations, expression of tau-RING prior to seeding or vice versa, monomeric tau was not affected. Expression of tau-RING constructs with mutations that render the RING domain inactive, as well as treatment with ubiquitination, VCP, or proteasome inhibitors, rescued the reduction of tau puncta in TVA cells, confirming that tau-RING acts through the same pathway as TRIM-away. The authors also demonstrated that the effect of tau-RING baits is tau ‘strain’ dependent. Wild-type tau-RING was shown to be less efficient than P301S tau-RING in degrading tau aggregates in TVA cells. In addition, 0N3R wild-type tau-RING and 0N4R wild-type tau-RING baits could degrade their respective tau isoform aggregates in HEK293-T cells expressing either 0N3R or 0N4R tau that were seeded with AD (preferentially 3R tau) [158] or PSP (4R tauopathy) brain-derived tau, respectively. AAV-mediated expression of a venus-P2A-tau-RING construct (VPTR) also protected Tg2541-derived, tau P301S primary neurons from seeded aggregation by ∼75 ​%. Lastly, intravenous injection of Tg2541 mice at 4 months with a brain targeting AAV-VPTR efficiently reduced the AT8 tau positive area measured by immunohistochemistry, reduced the amount of sarkosyl insoluble aggregated tau at 6 months, and prevented the age-associated decline in motor function.

#### Tau targeting intrabodies

Like TRIM-away, intrabodies are also an antibody-based method, but they function through a different mechanism. The term “intrabody” is a neologism derived from “intracellular” and “antibody”, referring to artificial intracellularly expressed antibodies [159]. Conceptualized in the late 80s, a proof-of-concept study showed the inactivation of a cytosolic enzyme by inducible expression of a full-length, enzyme-specific antibody in yeast [160]. A subsequent study optimized this approach by only expressing a single-chain variable fragment (scFv) in a fusion construct, combining the antigen-binding variable regions of the heavy and light chain of an antibody [161] targeting a human immunodeficiency virus type 1 (HIV-1) envelope protein, to develop an anti-viral therapeutic [162]. The first panel of scFv intrabodies that bind tau was reported in 2002 [163]. Due to a lack of the Fc region, intrabodies are not recognized by TRIM21 and only bind/sequester their target without actively inducing its degradation; hence, when unmodified, they are a form of immunotherapy, not TPD. To utilize intrabodies as a tool for TPD, the same group that published the first tau-binding intrabodies fused IkBα, a constitutive UPS substrate, to one of their anti-tau intrabodies, terming the method silencing intrabody technology (SIT; Fig. 5d) [164]. More recent approaches have designed bifunctional anti-tau scFv intrabodies fused with a ubiquitin tag for subsequent proteasomal or autophagic degradation [165], or a PEST degron sequence (sequence rich in proline (P), glutamic acid (E), serine (S) and threonine (T)) for direct, ubiquitin-independent, recognition by the proteasome [166]. These studies showed a significant reduction of wild-type and mutant tau in HEK cells overexpressing tau, primary neuronal cultures, P301S-htau mice, and FTD patient iPSC-derived 2D and 3D neuronal cultures. Interestingly, the intrabody with a ubiquitin tag designed to facilitate K48 polyubiquitination and proteasomal degradation was more effective in the mouse model than a tag leading to K63 polyubiquitination and autophagic degradation. While successful in a range of tauopathy model systems, the biggest challenge for intrabody-based TPD therapeutics is that they need to be expressed in the target cell population and, hence, need to be introduced as viral vector-based gene therapies.

### Other TPD approaches

As of the publication of this review, the aforementioned TPD approaches have been applied explicitly to tau degradation in a variety of model systems, but they are by far not an exhaustive list of all TPD methodologies. Additional TPD methods that have been employed against other proteins and targets could, in principle, be adapted to tau due to the modular nature of bifunctional molecules. In this section, we want to highlight a few of these approaches, including: AUTACs, ATTECs, MrTACs, and LYTACs.

#### AUTACs

Autophagy-targeting chimeras (AUTACs), together with ATTECs (discussed below), were the first autophagy-based degraders that directed POIs for autophagosomal degradation by tethering them to components of the ALP machinery. Developed in 2019, AUTACs mimic the S-guanylation of proteins via 8-nitrocyclic guanosine monophosphate (8-nitro-cGMP), a process that plays an important role in xenophagy, a selective type of autophagy responsible for the degradation of cytoplasm-invading bacteria [167]. Building on the discovery that S-guanylation of bacteria leads to its poly-ubiquitination and sequestration into autophagosomes for degradation [168], Arimoto and colleagues [169] developed bifunctional small molecules containing a *p*-fluorobenzylguanine (FBnG) that mimics S-guanylation while avoiding cGMP signaling (Fig. 3e). Combining the FBnG module with various ligands for targets of interest, the authors demonstrated efficient degradation of cytosolic proteins and mitochondria in a cellular system. While the autophagy receptor that recognizes the S-guanylation motif is still unknown, designing heterobifunctional AUTACs utilizing the FBnG module and a tau warhead could be a new avenue to direct this methodology toward targeted tau degradation.

#### ATTECs

In 2019, the groups of Boxun Lu and Yian Fei [170] reported on four compounds that bind to LC3B. This autophagosome membrane component interacts with SARs for selective autophagy, and with pathogenic proteins with expanded polyglutamine (polyQ) stretches like huntingtin (exon 1, polyQ72) and ataxin 3 (polyQ74). Shortly after, these compounds that interact with POIs and LC3B were named autophagosome-tethering compounds (ATTECs) [171]. These compounds could also be considered molecular glues [170] given that through binding to one protein, they promote change or stabilization of its tertiary structure, thereby increasing affinity for binding partners [172]. The initial four ATTECs were small (250–520 ​kDa), did not contain individual modules with distinct binding profiles, and could bind LC3B and polyQ-proteins independent of the presence of a second ligand warhead. Building on the identified LC3B binding compounds, the same group subsequently developed truly bifunctional ATTECs (Fig. 3f) that tethered lipid droplets [173] or mitochondria [174] to LC3B and directed them to degradation through the ALP. It stands to reason that combining the identified LC3B binding compounds with one of the available tau binders could be a way to develop tau-targeting ATTECs.

#### MrTACs

Methylarginine targeting chimeras are the newest addition to the repertoire of TPD modalities [175]. Degradation of target proteins is achieved by inducing proximity between the POI and protein arginine methyltransferases (PRMT). This induces arginine methylation on the POI that acts as a degron for lysosomal degradation through microautophagy [176]. In a proof of principle study, Seabrook et al. showed that bifunctional molecules that induce proximity between a PRMT1-Halo-tag construct and POI–SNAP-tag fusion proteins, as well as endogenous POIs with well-characterized binders (BRD4 and HDAC6), induced arginine methylation and subsequent lysosomal degradation of these targets [175]. A current roadblock to the broader application of MrTACs is the absence of “silent” PRMT ligands that could be used for designing bifunctional degraders that engage endogenous PRMTs for target degradation without affecting their endogenous function. Given the high level of research activity in the field of TPD approaches, this is likely achievable in the near future.

#### LYTACs

All the TPD approaches discussed so far act intracellularly, where the vast majority of disease-associated tau is found. However, as broadly investigated, removing the extracellular or cell-free fraction of tau oligomers or seeds, might be beneficial in tauopathies as a strategy to prevent re-seeding of native tau aggregation in neighboring cells and preventing pathology spreading. This could be achieved by lysosome-targeting chimeras (LYTACs) that direct extracellular or membrane-residing targets for degradation [177,178]. This takes advantage of the function of lysosome targeting receptors (LTRs). When cell surface residing LTRs bind their ligands, they are internalized together via endocytosis, dissociate in endosomes, and the ligand is directed toward lysosomal degradation while the LTR is recycled back to the plasma membrane [179] (Fig. 5e). LYTACs are small molecules, or more commonly antibodies, tagged with LTR ligands that enable their own endocytosis together with the cargo-target of interest by activating LTR lysosomal processing. While the warhead defines target specificity, the chosen glycosyl tag influences which LTR enables degradation. Some LTRs have tissue-specific expression, thereby enabling some specificity over tissue/cellular target selection and degradation. The first described LYTAC engages the broadly expressed cation-independent mannose-6-phosphate receptor with a mannose-6-phosphonate tag [178], whereas the second generation LYTAC binds the liver-specific asialoglycoprotein receptor through triantennary N-acetylgalactosamine [177]. With available binders and antibodies to disease-associated forms of tau, it will be interesting to investigate how tau-targeting LYTACs slow down the inter-cellular spreading of tau pathology in different tauopathy disease models.

## Alternative Technologies to TPD, Pros and Cons

Currently, the principal and most advanced strategies for targeting and reducing tau in tauopathies are immunotherapies (active and passive) and anti-sense oligonucleotides (ASOs). Of sixteen potential tau immunotherapies described in the literature, five have already been discontinued, and the remaining, as well as two types of ASOs, have entered at least phase I clinical trials [180,181]. Two of the challenges tau immunotherapies face are which tau species or epitope to target and the best delivery route. Currently, two active immunotherapies, AADvac1 and ACI-35.030, are in development and are essentially anti-pathological tau vaccines designed to elicit immune responses against disease-specific forms of tau. In clinical trials, both induced the production of *anti*-phospho-tau and anti-aggregated-tau specific antibodies, and AADvac1 also decreased cerebrospinal fluid (CSF) neurofilament light chain (NfL), a marker for neuroinflammation in neurodegenerative disorders [182]. Passive immunotherapies with monoclonal antibodies targeting a single protein epitope face additional challenges. Accessible epitopes in extracellular vs. intracellular pathological tau might differ [183], while both should likely be removed for optimal efficacy. Discontinued anti-tau antibodies failed to clear intracellular tau in preclinical assays [183], and the one that reached clinical trial was confirmed at postmortem analysis to have no impact on tau inclusions [184]. Since several studies show that neurons can take up antibodies [183], it seems unclear why these antibodies have failed to target intracellular tau, raising once again the question of what tau species or peptides are employed to generate the antibodies. In addition, passive immunotherapy treatment regimens currently necessitate intravenous infusions performed at a medical care facility, which requires many human and material resources, given the number of tauopathy patients, and invites complications and secondary effects that arise from immunotherapies in general.

Whereas tau immunotherapies target disease-relevant forms of tau by using antibodies that either bind specific phospho-epitopes or recognize misfolded oligomeric conformations, the two ASOs in development target *MAPT* expression to reduce total tau protein. Tau ASOs base pair with *MAPT* mRNA, forming a double-stranded ASO-mRNA complex that blocks ribosomal translation and is degraded by RNAse H [185]. Regarding delivery, ASOs are even harder to administer than passive immunotherapies because they do not transverse the blood-brain barrier and need to be injected intrathecally [185]. The early clinical trials for one of the tau ASOs in early-stage AD (BIIB080 developed by Biogen) did not show any serious adverse effects in patients and efficiently lowered CSF tau, which was used as a proxy for brain tau levels [186]. The next phases of its development will assess if significantly lowering CNS tau has therapeutic benefits without serious adverse effects.

While no tau-targeting TPD strategies have moved into the clinical stage yet, they have several attributes that set them apart from ASOs and immunotherapies. Due to their small molecule nature, optimizing blood-brain barrier penetrance will allow them to be formulated for simple oral delivery. Similarly, on the cellular level, small molecules can more readily enter the cytosol to interact with their target. Compared to biologics that need complicated and expensive production, small molecules can be readily synthesized at scale, have higher stability, and lower demands regarding storage. This would also allow for easier distribution in world regions with lower medical coverage or less developed medical infrastructures that are projected to suffer a stark increase in AD cases due to the demographic transition toward an “older population.” A potential drawback of TPD is that they make use of cell-intrinsic degradation mechanisms that have been shown in certain contexts to lose efficiency with age, to become disrupted in disease, and thus, to be affected in the same demographic that is also most likely to develop sporadic neurodegenerative diseases [116]. This will need to be addressed before starting clinical trials by potentially combining TPD therapeutics with therapeutic enhancers of the degradation pathway of choice. Adopting combination therapies is ultimately anticipated, as it has been done in other areas of medicine, such as cardiovascular disease and oncology.

## Outlook

The field of TPD is rapidly evolving with new modalities to target different proteins for degradation by cellular pathways, with publications occurring in fast succession. The combination of advances in structural biology, target-ligand design and modeling, PET probe development, and the ability to screen large chemical libraries for novel warheads against POIs and moieties that bind to UPS or ALP machinery components will enable the identification of ways to direct novel targets to degradation. ALP-based TPD technologies are still in their infancy compared to PROTACs, and more in-depth studies regarding their mechanism of autophagy engagement are needed to advance them toward drug development and pre-clinical stage. Anti-Aβ and tau immunotherapies have paved the way in supporting the hypothesis that the removal of pathogenic proteins in AD is disease-modifying and potentially even preventative, giving further validity to TPD as an approach for the treatment of AD. This is expected to also be beneficial to the field of tauopathies in general, as for all others, besides AD, no disease-modifying therapies are currently available, providing a clear area of unmet medical need that TPD is poised to address to realize the promise of precision medicine for neurodegeneration.

## Authors’ contributions

C. Alexander Sandhof: Conceptualization, Writing – Original Draft, Visualization.

Heide F. B. Murray: Writing – Original Draft.

M. Catarina Silva: Writing – Original Draft, Writing – review & editing.

Stephen J. Haggarty: Writing – review & editing.

## Funding

Funding for the Chemical Neurobiology Laboratory's neurodegenerative research has been provided by the National Institute of Health, Tau Consortium (Rainwater Charitable Foundation), the Association for Frontotemporal Dementia, CurePSP, the Stuart and Suzanne Steele MGH Research Scholars Program, and the Ludwig Neurodegenerative Disease Program at Harvard Medical School.

## Declaration of competing interest

The authors declare the following financial interests/personal relationships which may be considered as potential competing interests: M. Catarina Silva reports a relationship with Proximity Therapeutics that includes: consulting or advisory. M. Catarina Silva reports a relationship with Casma Therapeutics that includes: funding grants. Stephen J. Haggarty reports a relationship with Proximity Therapeutics that includes: board membership. Stephen J. Haggarty reports a relationship with Psy Therapeutics that includes: board membership. Stephen J. Haggarty reports a relationship with Birdwood Therapeutics that includes: board membership. Stephen J. Haggarty reports a relationship with Frequency Therapeutics Inc that includes: board membership. Stephen J. Haggarty reports a relationship with Souvien Therapeutics that includes: board membership. Stephen J. Haggarty reports a relationship with Sensorium Therapeutics that includes: board membership. Stephen J. Haggarty reports a relationship with 4 ​M Therapeutics that includes: board membership. Stephen J. Haggarty reports a relationship with Ilios Therapeutics that includes: board membership. Stephen J. Haggarty reports a relationship with Entheos Labs that includes: board membership. Stephen J. Haggarty reports a relationship with Amgen that includes: consulting or advisory and speaking and lecture fees. Stephen J. Haggarty reports a relationship with AstraZeneca that includes: consulting or advisory, funding grants, and speaking and lecture fees. Stephen J. Haggarty reports a relationship with Biogen that includes: consulting or advisory and speaking and lecture fees. Stephen J. Haggarty reports a relationship with Merck that includes: consulting or advisory and speaking and lecture fees. Stephen J. Haggarty reports a relationship with Regenacy Pharmaceuticals that includes: consulting or advisory and speaking and lecture fees. Stephen J. Haggarty reports a relationship with Syros Pharmaceuticals that includes: consulting or advisory and speaking and lecture fees. Stephen J. Haggarty reports a relationship with Juvenescence Life that includes: consulting or advisory and speaking and lecture fees. Stephen J. Haggarty reports a relationship with JW Pharmaceuticals that includes: funding grants. Stephen J. Haggarty reports a relationship with Lexicon Pharmaceuticals that includes: funding grants. Stephen J. Haggarty reports a relationship with Vesigen Therapeutics that includes: funding grants. Stephen J. Haggarty reports a relationship with Compass Pathways that includes: funding grants. Stephen J. Haggarty reports a relationship with Atai Life Sciences that includes: funding grants. Stephen J. Haggarty reports a relationship with Stealth Biotherapeutics that includes: funding grants. Stephen J. Haggarty, M. Catarina Silva has patent Compounds for Tau Protein Degradation licensed to Proximity Therapeutics. Stephen J. Haggarty, M. Catarina Silva has patent #Targeted Degrader of Aberrant Tau Based on the PET Tracer PBB3 pending to General Hospital Corp, Dana Farber Cancer Institute Inc. Stephen J. Haggarty, M. Catarina Silva has patent #Inhibitors of TTBK1 pending to General Hospital Corp, Dana Farber Cancer Institute Inc. If there are other authors, they declare that they have no known competing financial interests or personal relationships that could have appeared to influence the work reported in this paper.
